# Presence of dynapenia and association with anthropometric variables in cancer patients

**DOI:** 10.1186/s12885-020-07519-4

**Published:** 2020-10-19

**Authors:** Ana Beatriz Rechinelli, Isabele Lessa Marques, Eduarda Cristina Rodrigues de Morais Viana, Isadora da Silva Oliveira, Vanusa Felício de Souza, Glenda Blaser Petarli, Jose Luiz Marques Rocha, Valdete Regina Guandalini

**Affiliations:** 1 Nutrition Course. Department of Integrated Health Education, Federal University of Espirito Santo, Vitoria, Espırito Santo, Brazil. Marechal Campos avenue, 1468 – Maruípe, Vitória, Espírito Santo CEP: 29040-090 Brazil; 2 University Cassiano Antonio Moraes Hospital, Vitoria, Espırito Santo, Brazil. Marechal Campos avenue, 1355 – Santa Cecília, Vitória, Espírito Santo CEP: 29043-260 Brazil; 3 Postgraduate Program in Nutrition and Health, Federal University of Espirito Santo, Vitoria, Espırito Santo, Brazil. Avenida Marechal Campos avenue, 1468 – Maruípe, Vitória, Espírito Santo CEP: 29040-090 Brazil

**Keywords:** Dynapenia, Body composition, Muscle strength, Muscle mass, Cachexia

## Abstract

**Background:**

Dynapenia is defined as an age-related loss of muscle strength. There is little information on dynapenia in cancer patients and on how it relates to anthropometric variables. The aim of this study was to analyze the presence of dynapenia and its association with anthropometric variables in hospitalized cancer patients.

**Methods:**

Participants comprised adult and elderly cancer patients evaluated within the first 48 h of hospital admission to a tertiary public hospital, a referral center for gastrointestinal tract surgery. Anthropometric variables were measured according to standardized protocols. Dynapenia was identified based on handgrip strength (HGS), according to the cutoff points defined by the European Working Group on Sarcopenia in Older People (EWGSOP2), with values for women < 16 kg and for men < 27 kg. Statistical analysis was performed using SPSS software, version 22.0, with a significance level of 5%.

**Results:**

This study included 158 patients aged in average 59.5 ± 14.0 years; of these, 53.6% were elderly, 58.9% non-white and 59.5% had some degree of malnutrition. The most prevalent type of cancer was that of the lower gastrointestinal tract (33.5%). The presence of dynapenia was observed in 23.4% of the patients and cachexia in 36.1%. There was an association between dynapenia with age (*p* < 0.001), life stage (*p* = 0.002) and race/color (*p* = 0.027), and also with body mass index (BMI) (*p* = 0.001) and adductor pollicis muscle thickness (APMT) of both hands (*p* < 0.05). After logistic regression analysis, adjusted for the sociodemographic variables, the APMT of the dominant hand and the low weight determined by body mass index remained associated with the occurrence of dynapenia (*p* < 0.05).

**Conclusions:**

In this study we confirmed that dynapenia was present in cancer patients, being associated with APMT of the dominant hand and low weight. HSG was proven to be a reliable and complementary measure to be added to the process of assessing nutritional status, contributing to the nutritional diagnosis of these patients and to the detection of early muscle depletion.

## Background

The prevalence of malnutrition in cancer patients varies from 31 to 97% [1, 2] according to the location of the tumor, stage of the disease, and type of anticancer treatment adopted [3]. Patients with gastrointestinal tract cancer are the most affected, for about 15 to 66% of them are malnourished [3, 4].

Malnutrition is associated with a worse prognosis, the presence of complications and a reduced response to treatment, in addition to increasing the length of hospital stay and being an independent factor for shorter survival [4–6]. Anorexia is often observed, along with a rapid loss of adipose tissue, tissue loss, and skeletal muscle atrophy [7], which may or may not progress to cachexia [8].

Cancer cachexia is a multifactorial syndrome known for involuntary weight loss concomitant with reduced skeletal muscle mass, with or without loss of adipose tissue, being associated with decreased function, physical performance and a negative prognosis [8, 9]. It happens as a result of a negative energy-protein balance, reduced food intake and an increased catabolic process, being aggravated by the systemic inflammation syndrome that often takes place in cancer patients [8–12]. The worsening of the cachectic condition leads to a progressive impairment of muscle function, which may explain its association with dynapenia in these patients [13].

Dynapenia is defined as an age-related loss of strength and muscle function [14], having been considered an important predictor of functional disability and death related to the loss of isolated muscle mass [15, 16]. Its development varies according to the individuals’ pre-existing characteristics, such as nutritional status, body composition, physical activity, food intake, and comorbidities. When present simultaneously, these factors may lead to loss of muscle mass and, consequently, to the worsening of nutritional status [15].

Some studies have shown that dynapenia is more prevalent than sarcopenia [13, 17], which prompted an interest in investigating the relationship between muscle function, nutritional status and body composition.

The measurement of handgrip strength (HGS), which can be used to determine dynapenia, has been shown to reflect nutritional impairment even before changes in body composition could be detected through different anthropometric parameters that indicate muscle mass reserve [18, 19], such as the adductor pollicis muscle thickness (APMT), mid-arm circumference (MAC), corrected mid-upper arm muscle area (CAMA) and calf circumference (CC), all commonly used for the assessment of nutritional status and body composition in cancer patients [20, 21].

However, there is still a lack of evidence on how these measures are actually interrelated. A better understanding of this relationship would provide support for the assessment of more effective, practical and easier ways to diagnose the nutritional status of hospitalized patients earlier. Therefore, the aim of this study was to analyze the presence of dynapenia and its association with anthropometric variables in hospitalized cancer patients.

## Methods

### Design

This descriptive cross-sectional study was carried out in a public tertiary hospital – a referral center for gastrointestinal tract surgery – located in Vitória-ES, Brazil, from March 2017 to October 2019.

### Study population

The study included patients of both sexes, aged 20 years or over, with a confirmed diagnosis of cancer, admitted to the General Surgery Unit to perform a curative surgical procedure to remove the neoplastic tumor. The recruitment was carried out by trained researchers, and, at first, all eligible patients were approached. Individuals with cognitive dysfunction predicted in medical records, in respiratory isolation, in palliative care, with full body edema or those who could not be evaluated by the researchers within the first 48 h of hospital admission were excluded.

Tumor location was categorized as follows: upper gastrointestinal tract (GIT), lower GIT, attached glands (liver, pancreas, and bile ducts), and others (lung and chest, hematological, skin, unknown behavior, ovary, gastrointestinal stroma, bladder, cervical, ganglia, parotid gland, breast, mediastinum, peritoneum, pleura, connective tissue, thyroid, submandibular and other locations). The length of hospital stay was categorized as follows: less than or equal to 7 days of hospitalization and greater than 7 days, according to the calculated median.

### Study variables

Trained researchers applied specific protocols in order to collect sociodemographic (age, sex, and self-declared color) and anthropometric information, such as weight (kg), height (m), handgrip strength (HGS) (kg/f), arm circumference (AC) (cm), tricipital skinfold (TSF) (mm), calf circumference (CC) (cm) and thickness of the adductor pollicis muscle thickness (APMT) (mm), considered indicators of muscle mass and body composition [22–25]. Mid-arm circumference (MAC) (cm) and corrected mid-upper arm muscle area (CAMA) (cm^2^), which also evaluate the muscular compartments, were calculated from AC and TSF [22, 26]. All anthropometric variables were measured according to standardized protocols [27, 28].

For the classification of AC, TSF and MAC, we used the adequacy to the 50th percentile (%), according to Frisancho et al. Values < 90% were considered low and ≥ 90% adequate. For the classification of CAMA, values lower than the 15th percentile, according to sex and age, were considered reduced [22]. CC was classified according to the cutoff points proposed by Barbosa-Silva et al., who defined values ≤33 cm for women and ≤ 34 cm for men [24].

Body mass index (BMI) was calculated (weight (kg)/height^2^ (m)) and classified according to the World Health Organization (WHO) [29] reference for adults, and to that proposed by the Pan American Health Organization (PAHO) [30] for the elderly. In the present study, the term “overweight” refers to individuals classified as overweight or obese, according to the BMI.

To identify cachexia, the patients were classified according to the following diagnostic criteria: (a) unintentional weight loss ≥5% in the last 6 months or (b) unintentional weight loss ≥2% in the last 6 months and BMI < 20 kg/m^2^ [9].

APMT was measured according to the technique proposed by Lameu et al. [23] using a Lange® adipometer. The procedure was performed three times on both dominant (DAPMT) and non-dominant (NDAPMT) hands, and the mean value considered for the analysis [31]. The cutoff point of Bragagnolo et al. was used for surgical patients, with measures lower than 13.4 mm for the DAPMT and 13.1 mm for the NDAPMT indicating malnutrition [31].

To assess HGS we used the Jamar® handheld dynamometer. The test was performed according to the methodology recommended by the American Hand Therapy Association (ASHT) [32]. The procedure was performed three times on the dominant hand (DHGS) with maximum effort for about 5 s, with an interval of 1 min between measurements [32]. The outcome variable “dynapenia” was identified from the maximum HGS of the dominant hand, according to the cutoff points defined by the European Working Group on Sarcopenia in Older People (EWGSO), with values of < 16 kg for women and < 27 kg for men [33].

The Patient-Generated Subjective Global Assessment (PG-SGA) was collected through the application of a questionnaire translated and validated into Portuguese by Gonzales et al. [34], with permission to use the PG-SGA/Pt-Global Platform (www.pt-global.org) having been duly obtained. The PG-SGA is the subjective nutritional assessment and screening tool indicated by the Brazilian Consensus of Oncological Nutrition for the evaluation of cancer patients in Brazil [18]. It allows the classification of nutritional status into three categories: (A) well nourished, (B) suspected or moderate malnutrition, and (C) severe malnutrition.

### Statistical analysis

Descriptive analysis was expressed in means and standard deviations to describe continuous variables and in percentages for categorical variables. Patients were grouped into adults (< 60 years old) and elderly (≥ 60 years old), according to the WHO classification for developing countries [35]. Race/color was grouped into whites and non-whites. Differences between the proportions were assessed by the Chi-square test. To determine the influence of independent variables in the presence of dynapenia (dependent variable) and anthropometric variables (independent ones), binary logistic regression analysis was performed. Crude Odds Ratios (ORs) were presented after adjustments for sociodemographic variables (sex, age range and race/color). The adjustment variables were inserted in blocks: model 1 (anthropometric variables and age), model 2 (anthropometric variables, age and sex) and model 3 (anthropometric variables, age, sex, and race/color) and model 4 (anthropometric variables, age, sex, color, malnutrition and cachexia). Only anthropometric variables with 5% statistical significance were selected for logistic regression in the chi-square test and that could have an influence on muscle strength (malnutrition and cachexia). Data were analyzed using SPSS 22.0 software. The level of significance adopted for all tests was 5.0%.

## Results

A total of 158 patients aged in average 59.5 ± 14.0 years were evaluated. Of these, 56.3% were elderly, 51.3% were male, and 58.9% declared themselves non-white. As for tumor location, those on the lower gastrointestinal tract (GIT) were more prevalent, affecting 33.5% of patients. Most patients remained hospitalized for ≤7 days (54.4%). PG-SGA revealed that 59.5% of patients had some degree of malnutrition (B + C). Moreover, cachexia was detected in 36.1% of the patients, while 23.4% presented dynapenia (Table 1).

**Table 1:** Sociodemographic characteristics, tumor location, length of hospital stay, nutritional status and presence of cachexia and dynapenia in hospitalized cancer patients

Variable (***n*** = 158)	Total
**Age** (mean ± SD)	59.5 ± 14.0
**Min-Max**	20–88
**Stage of life**	**n (%)**
Adult	69 (43.7)
Elderly	89 (56.3)
**Gender**	
Male	81 (51.3)
Female	77 (48.7)
**Color**	
Non-white	93 (58.9)
White	65 (41.1)
**Tumor Location**	
Lower GIT	53 (33.5)
Attached glands	39 (24.7)
Upper GIT	27 (17.1)
Others^a^	39 (24.7)
**Length of hospital stay**	
≤ 7 days	86 (54,4)
> 7 days	72 (45,6)
**PG-SGA**	
Well nourished (A)	64 (40.5)
Moderately malnourished (B)	64 (40.5)
Severely malnourished (C)	30 (19.0)
**Presence of Cachexia**	
Yes	57 (36.1)
No	101 (63.9)
**Dynapenia**	
Yes	37 (23.4)
No	121 (76.6)

^a^Others (corresponding to cancers with a low prevalence within the sample): 6.33%: lung and thorax cancer; 3.80%: hematological cancer; 3.16%: skin cancer; 1.89%: cancer of unknown behavior; 1.26%: ovarian cancer; 0.63%: stromal gastrointestinal cancer; 0.63%: bladder cancer; 0.63%: cervical cancer; 0.63%: ganglion cancer; 0.63%: parotid gland cancer; 0.63%: breast cancer; 0.63%: mediastinal cancer; 0.63%: cancer from other locations; 0.63%: peritoneum cancer; 0.63%: pleural cancer; 0.63%: connective tissue cancer; 0.63%: thyroid cancer; 0.63%: submandibular cancer. PG-SGA: Patient-Generated Subjective Global Assessment

We found significant differences between the presence and absence of dynapenia in relation to some sociodemographic variables, namely age (*p* < 0.001), stage of life (*p* = 0.002) and race/color (*p* = 0.027) (Table 2).

**Table 2:** Distribution of sociodemographic variables, tumor location, length of stay, nutritional status and cachexia according to the categories of dynapenia in hospitalized cancer patients

Variables (***n*** = 158)	Without dynapenia	With dynapenia	*p* value
**Age** (mean ± SD)	57.4 ± 13.6	66.6 ± 13.3	**< 0.001**
	**n (%)**	**n (%)**	
**Stage of life**			**0.002**
Adult	61 (88.4)	8 (11.6)	
Elderly	60 (67.4)	29 (32.6)	
**Gender**			0.130
Male	58 (71.6)	23 (28.4)	
Female	63 (81.8)	14 (18.2)	
**Color**			**0.027**
Non-white	77 (82.8)	16 (17.2)	
White	44 (67.7)	21 (32.2)	
**Tumor location**			0.566
Lower GIT	40 (75.5)	13 (24.5)	
Attached glands	28 (7.8)	11 (28.2)	
Upper GIT	20 (74.1)	7 (25.9)	
Others^a^	33 (84.6)	6 (15.4)	
**Length of hospital stay**			0.236
≤ 7 days	69 (80.2)	17 (19.8)	
> 7 days	52 (72.2)	20 (27.8)	
**PG-SGA**			0.308
Well nourished (A)	46 (71.9)	18 (28.1)	
Moderately malnourished (B)	53 (82.8)	11 (17.2)	
Severely malnourished (C)	22 (73.3)	8 (26.7)	
**Cachexia**			0.518
Yes	79 (78.2)	22 (21.8)	
No	42 (73.7)	15 (26.3)	

GIT: gastrointestinal tract. Student’s t-test; ^a^Others (corresponding to cancers with a low prevalence within the sample): 6.33%: lung and thorax cancer; 3.80%: hematological cancer; 3.16%: skin cancer; 1.89%: cancer of unknown behavior; 1.26%: ovarian cancer; 0.63%: stromal gastrointestinal cancer; 0.63%: bladder cancer; 0.63%: cervical cancer; 0.63%: ganglion cancer; 0.63%: parotid gland cancer; 0.63%: breast cancer; 0.63%: mediastinal cancer; 0.63%: cancer from other locations; 0.63%: peritoneum cancer; 0.63%: pleural cancer; 0.63%: connective tissue cancer; 0.63%: thyroid cancer; 0.63%: submandibular cancer. PG-SGA: Patient-Generated Subjective Global Assessment

Table 3 shows the association between anthropometric variables and the categories of dynapenia. We found the presence of dynapenia to be significantly affected by BMI (p < 0.001), DAPMT (p < 0.001) and NDAPMT (p = 0.002) (Table 3).

**Table 3:** Association between stage of life and anthropometric variables according to the categories of dynapenia in hospitalized cancer patients

Variables (n = 158)	Without dynapenia	With dynapenia	p value
	n (%)	n (%)	
**BMI**			**< 0.001**
Low weight	25 (52.1)	23 (47.9)	
Eutrophic	45 (84.9)	8 (15.1)	
Overweight	51 (89.5)	6 (10.5)	
**CC**			0.706
Reduced	68 (78.2)	19 (21.8)	
Adequate	53 (74.6)	18 (25.4)	
**AC**			1.000
Reduced	56 (76.7)	17 (23.3)	
Adequate	65 (76.5)	20 (23.5)	
**TSF**			0.850
Reduced	52 (75.4)	17 (24.6)	
Adequate	69 (77.5)	20 (22.5)	
**MAC**			0.453
Reduced	71 (78.9)	19 (21.1)	
Adequate	50 (73.5)	18 (28.5)	
**CAMA**			0.258
Reduced	75 (79.8)	19 (20.2)	
Adequate	46 (71.9)	18 (28.1)	
**DAPMT**^**a**^			**< 0.001**
Reduced	55 (65.5)	28 (33.7)	
Adequate	64 (90.1)	7 (9.9)	
**NDAPMT**^**b**^			**0.007**
Reduced	62 (69.7)	27 (30.3)	
Adequate	57 (87.7)	7 (12.3)	

GIT: gastrointestinal tract. Chi-square test. *BMI* Body mass index, *CC* Calf circumference, *AC* Arm circumference, *TSF* Tricipital skinfold, *MAC* Mid-arm muscle circumference, *CAMA* Corrected mid-upper arm muscle area, *DAPMT* dominant adductor pollicis muscle thickness, *NDAPMT* Non-dominant adductor pollicis muscle thickness; ^a^n = 154; ^b^n = 154

In the logistic regression analysis, even after adjusting for age, sex and race/color, nutritional status by PG-SGA and cachexia, DAPMT and low weight remained associated with the occurrence of dynapenia. In relation to DAPMT, patients with reduced musculature were 5.54 times more likely to have dynapenia compared to individuals with adequate APMT (OR: 5.54 [95% CI: 1.04–29.39], *p* = 0.044). When analyzing by BMI, we found that those with low weight were approximately 4.55 times more likely to have dynapenia compared to eutrophic patients (OR: 4.55 [95% CI 1.47–14.08], *p* = 0.009). Despite the inclusion of malnutrition and cachexia in the regression model, we observed that DAPMT and low weight (BMI) remained associated with dynapenia. However, NDAPMT and overweight lost the strength of the association after adjusted analyses (Table 4).

**Table 4:** Logistic regression models for dynapenia in hospitalized cancer patients

	Crude OR (IC95%)	Model 1 OR (IC95%)	Model 2 OR (IC95%)	Model 3 OR (IC95%)	Model 4 OR (IC95%)
**DAPMT (mm)**					
Adequate	1	1	1	1	1
Reduced	**4.65 (1.88–11.48)**	**4.70 (1.03–22.0)**	**5.13 (1.05–25.10)**	**5.86 (1.10–31.06)**	**5.54 (1.04–29.39)**
**NDAPMT (mm)**					
Adequate	1	1	1	1	1
Reduced	**3.10 (1.30–7.38)**	1.22 (0.26–5.62)	1.14 (0.24–5.44)	0.84 (0.15–4.49)	1.02(0.20–5.30)
**BMI (kg/m**^**2**^**)**					
Eutrophy	**1**	1	1	1	1
Low weight	**5.17 (2.02–13.26)**	**5.66 (2.01–3.31)**	**4.17 (1.42–12.25)**	**4.83 (1.58–14.71)**	**4.55(1.47–14.08)**
Overweight	**0.66 (0.21–2.05)**	1.00 (0.30–3.31)	1.14 (0.24–5.44)	1.02 (0.30–3.47)	1.12(0.32–3.94)

Model 1: adjusted for gender. Model 2: adjusted for gender and stage of life. Model 3: adjusted for gender, stage of life and color Model 4: adjusted for gender, stage of life, color, malnutrition (PG-SGA) and cachexia. *BMI* Body mass index, *DAPMT* dominant adductor pollicis muscle thickness, *NDAPMT* Non-dominant adductor pollicis muscle thickness. The numbers in bold indicate those where p < 0.05

## Discussion

This study identified dynapenia in 23.4% of the evaluated patients, who were mostly malnourished and had gastrointestinal tract cancer. Similar studies carried out in both adults and the elderly showed that dynapenia was present in 24.4% [36] and 44.9% [37] of cancer patients, respectively, while yet another study reported that 30.9% of the elderly population under evaluation presented dynapenia [13].

The incidence of malnutrition varies between 31 to 97% in cancer patients [1, 2], being more prevalent in cancers located in the GIT due to obstructions in this region and/or malabsorption of nutrients [38].

The metabolic changes resulting from cancer, physical inactivity, the adverse effects of treatment, and the presence of malnutrition, impair the maintenance of muscle mass and function, causing functional impairments, total physical disability and mortality [14, 38]. The impairment of the muscle’s ability to generate strength leads to dynapenia, which further aggravates clinical and nutritional aspects [13]. In our study the presence of malnutrition and cachexia did not influence dynapenia.

Dynapenia can be diagnosed through the HGS, a measure of functional capacity that complements the nutritional assessment for being associated with malnutrition [39–41], in addition to being recommended by the American Society for Parenteral and Enteral Nutrition (ASPEN) for the diagnosis of malnutrition in clinical practice [42].

Magnetic resonance imaging, computed tomography, biphotonic X-ray absorptiometry (DXA) and electrical bioimpedance are excellent methods for the estimation of muscle mass, for they allow the precise quantification of muscle tissue and their use in research has increased the understanding of body composition [43]. This such methods are expensive, and only a few are accessible to clinical practice [36]. Thus, it is necessary to investigate tools capable of early identification of nutritional risk and possible changes in body composition, given the implications for the response to treatment, permanence and clinical outcomes in cancer patients [33].

In hospital practice, anthropometry is used to identify the risk and nutritional status of each patient and to assess the most appropriate nutritional and clinical interventions [21]. Despite the practicality of measurement, the use of HGS for such assessments is still limited. In this study, the presence of dynapenia was associated only with reduced values of DAPMT and BMI, even in the model adjusted for gender, stage of life and color/race. As for the other anthropometric variables studied here, those appear to depend on a more advanced picture of muscle impairment and on an association between different measures to yield results, depending also on the measurement techniques, the patient’s hydration status and applied equations.

Pereira et al., when evaluating 100 cancer patients in outpatient treatment, found an association of HGS with MAC and a weak correlation with CAMA only in the left hand. However, regression models were not used then, nor were cut-off points considered for HGS. The authors confirm the importance of using HGS as an auxiliary method in nutritional screening and as a complementary method in the nutritional diagnosis of cancer patients, in addition to the need for further studies [41].

As for the adductor pollicis muscle, it is the only muscle that can be directly measured by anthropometry, having been established as a good predictor of muscle mass loss [23, 44]. The measurement of APMT has been rather efficiently employed in the evaluation of the muscular compartment, being associated with muscle mass reduction, malnutrition and mortality [45–47]. Valente et al., observed significant correlations between APMT, anthropometric measurements and nutritional status, according to PG-SGA, in adult and elderly cancer patients [25].

Guerra et al. observed a positive correlation between HGS and APMT values in adult hospitalized patients [40], which demonstrates a relationship between such measures and the ability to assess strength and muscle mass.

The literature recognizes HGS as a sensitive, low-cost and easy-to-use method to assess functional muscle loss and nutritional risk [48, 49]. The present results suggest that it is also efficient for assessing muscle mass loss in cancer patients, both in adults and the elderly.

In cancer patients, lower HGS values are associated with unfavorable clinical outcomes, such as longer hospital stays, poor quality of life, postoperative complications and mortality [33, 36, 50]. However, the measure is still seldom used in hospital and outpatient clinical practice, as there are no specific cutoff points for cancer patients and for the evaluation of reduced HGS values in non-elderly subjects [39, 50].

The weight loss and reduced lean mass seen in cancer patients are associated with decreased food intake and the systemic inflammation syndrome induced by the presence of the tumor, which leads to increased pro-inflammatory cytokine activity [10, 11]. It also induces anabolic resistance, which decreases protein synthesis. Combined, all these mechanisms result in weight loss and lean mass depletion [8].

In the elderly, the mechanisms related to changes in muscle tissue may be attributed to a combination of neural factors, such as impaired neural activation and failures in neuromuscular and muscle transmission, accentuating the loss of muscle mass and strength with advancing age in cancer patients [14, 51].

Thus, identifying the presence of dynapenia in adult patients can reduce changes that would be enhanced by aging, which is confirmed by the association between dynapenia and stage of life: HGS was lower in elderly patients. Therefore, the interpretation of these results are limited by the absence of specific cutoff points for the classification of dynapenia in adults and in cancer patients.

Dynapenia is still a new concept that needs to be further investigated, especially in cancer patients. It is known that the loss of muscle mass affects 80% of these patients; however, that cannot be interpreted aside from muscle function, which is reflected by muscular strength [36].

A study conducted by Moreau et al. with 150 cancer patients revealed that the loss of muscle mass, assessed by computed tomography, was greater than the presence of dynapenia, what calls for further studies to elucidate the diagnostic criteria, the chronology of dynapenia and the loss of muscle mass, especially in this population [50].

There are no studies associating the presence of dynapenia with anthropometric variables in cancer patients, what also limits this discussion. In addition, as the present study was carried out in a referral center for surgical rather than antineoplastic treatment, tumor staging was not available for most patients, which hindered the conduction of analyses addressing this variable in relation to the presence of dynapenia.

As a contribution, our study demonstrates clinical relevance in investigating dynapenia in cancer patients, both in adults and the elderly, adding to the knowledge on the subject. We believe our results stimulate further studies, and that definitions and cut-off points can be established with better precision.

## Conclusion

In this study, the presence of dynapenia in cancer patients was confirmed through HSG, being associated with DAPMT and low weight. HGS was proven to be a reliable measure to be added – along with the other relevant variables – to the nutritional status assessment process, potentially contributing to the nutritional diagnosis of these patients and to the early detection of muscle depletion. However, it is still necessary to better elucidate dynapenia in cancer patients at different stages of life.

## Data Availability

The data that support the findings of this study are available from the corresponding author on reasonable request.

## Abbreviations

AC: Arm circumference
ASPEN: American Society for Parenteral and Enteral Nutrition
ASTH: American Hand Therapy Association
BIA: Electrical bioimpedance
BMI: Body mass index
CAMA: Corrected mid-upper arm muscle area
CC: Calf circumference
DAPMT: Dominant adductor pollicis muscle thickness
DXA: Dual energy x-ray absorptiometry
EWGSO: European Working Group on Sarcopenia in Older People
GIT: Gastrointestinal tract
HGS: Handgrip strength
MAC: Mid-arm circumference
NDAPMT: Non-dominat adductor pollicis muscle thickness
PG-SGA: Patient-Generated Subjective Global Assessment
TSF: Tricipital skinfold
TAPM: Thickness of the adductor pollicis muscle
WHO: World Health Organization

## References

[1] Ge T, Lin T, Yang J, Wang M (2019). Nutritional status and related factors of patients with advanced lung cancer in northern China: a retrospective study. Cancer Manag and Res.

[2] Hébuterne X, Lemarié E, Michallet M, de Mentreuil CB, Schneider SM, Goldwasser F (2014). Prevalance of malnutrition and current use of nutritional support in patients with cancer. JPEN J Parenter Enteral Nutr.

[3] Correia MITD, Perman MI, Waitzberg DL (2017). Hospital malnutrition in Latin America: a systematic review. Clin Nutr.

[4] Na BG, Han SS, Cho YA (2018). Nutritional status of patients with Cancer: a prospective cohort study of 1,588 hospitalized patients. NutrCancer..

[5] Nishiyama VKG, Albertini SM, Moraes CMZG, Godoy MF, Netinho JG (2018). Malnutrition and clinical outcomes in surgical patients with colorectal disease. ArqGastroenterol..

[6] Planas M, Álvarez-Hernández J, Léon-Sanz M (2016). Prevalence of hospital malnutrition in cancer patients: a sub-analysis of the PREDyCES® study. Support Care Cancer.

[7] Kabata P, Jastrzebski T, Kakol M (2015). Preoperative nutritional support in cancer patients with no clinical signs of malnutrition -- prospective randomized controlled trial. Support Care Cancer.

[8] Arends J, Bachmann P, Baracos V (2017). ESPEN guidelines on nutrition in cancer patients. Clin Nutr.

[9] Fearon K, Strasser F, Anker SD (2011). Definition and classification of cancer cachexia: an international consensus. Lancet Oncol.

[10] Garofolo A, Petrilli AS (2006). Omega-3 and 6 fatty acids balance in inflammatory response in patients with cancer and cachexia. Rev Nutr.

[11] Aoyagi T, Terracina KP, Raza A, Matsubara H, Takabe K (2015). Cancer cachexia, mechanism and treatment. World J Gastrointest Oncol.

[12] Souza NCS, Gonzalez MC, Martucci RB (2019). Comparative analysis between computed tomography and surrogate methods to detect low muscle mass among colorectal Cancer patients. JPEN J Parenter Enteral Nutr.

[13] Alexandre TDS, Duarte YAO, Santos JLF, Lebrão ML (2018). Prevalence and associated factors of sarcopenia, dynapenia, and sarcodynapenia in community-dwelling elderly in São Paulo - SABE Study. Rev Bras Epidemiol..

[14] Clark BC, Manini TM (2010). Functional consequences of sarcopenia and dynapenia in the elderly. CurrOpin Clin NutrMetab Care.

[15] Clark BC, Manini TM (2012). What is dynapenia?. Nutrition..

[16] Newman AB, Kupelian V, Visser M (2006). Strength, but not muscle mass, is associated with mortality in the health, aging and body composition study cohort. J Gerontol A Biol Sci Med Sci.

[17] Toyoda H, Hoshino M, Ohyama S (2019). Impact of sarcopenia on clinical outcomes of minimally invasive lumbar decompression surgery. Sci Rep.

[18] Consenso Nacional de Nutrição Oncológica (2016). Instituto Nacional de Câncer. Ministério da Saúde. 2a ed.

[19] Schlüssel MM, dos Anjos LA, de Vasconcellos MT, Kac G (2008). Reference values of handgrip dynamometry of healthy adults: a population-based study. Clin Nutr.

[20] Madden AM, Smith S (2016). Body composition and morphological assessment of nutritional status in adults: a review of anthropometric variables. J Hum Nutr Diet.

[21] Mendes J, Afonso C, Moreira P (2019). Association of Anthropometric and Nutrition Status Indicators with hand grip strength and gait speed in older adults. JPEN J Parenter Enteral Nutr.

[22] Frisancho AR (1981). New norms of upper limb fat and muscle areas for assessment of nutritional status. Am J Clin Nutr.

[23] Lameu EB, Gerude MF, Corrêa RC, Lima KA (2004). Adductor pollicis muscle: a new anthropometric parameter. RevHospClinFacMedSao Paulo.

[24] Barbosa-Silva TG, Bielemann RM, Gonzalez MC, Menezes AM (2016). Prevalence of sarcopenia among community-dwelling elderly of a medium-sized South American city: results of the COMO VAI? study [published correction appears in J Cachexia Sarcopenia Muscle. 2016 Sep;7(4):503]. J Cachexia Sarcopenia Muscle.

[25] Valente KP, Almeida BL, Lazzarini TR (2019). Association of Adductor Pollicis Muscle Thickness and Handgrip Strength with nutritional status in cancer patients. PLoS One.

[26] Frisancho AR (1990). Anthropometric standards for the assessment of growth and nutritional status.

[27] World Health Organization (1995). Physical status: the use and interpretation of anthropometry - report of a WHO expert committee - WHO technical report series 854.

[28] Lohman TG, Roche AF, Martorell R (1988). Anthropometric standardization reference manual.

[29] World Health Organization. Physical Status: The Use And Interpretation of Anthropometry - Report of a WHO Expert Committee - WHO Technical Report Series 854. Geneva: WHO Expert Committee on Physical Status; 2000. 8594834

[30] OPAS. Organização Pan-Americana. XXXVI Reunión Del Comitê Asesor de Investigaciones em Salud – Encuestra Multicêntrica – Salud Beinestar y Envejecimeiento (SABE) em América Latina e el Caribe. 2002. Available in: http://www.opas.org/program/sabe.htm. Accessed 20 July 2020.

[31] Bragagnolo R, Caporossi FS, Dock-Nascimento DB, de Aguilar-Nascimento JE (2009). Adductor pollicis muscle thickness: a fast and reliable method for nutritional assessment in surgical patients. Rev Col Bras Cir.

[32] Fess EE (1992). Clinical Assessment recommendations, 2nd ed. Garner, nc: American Society of Hand Therapists (asht, ed.).

[33] Cruz-Jentoft AJ, Bahat G, Bauer J (2019). Sarcopenia: revised European consensus on definition and diagnosis. Age Ageing.

[34] Gonzalez MC, Borges LR, Silveira DH, Assunção MCF, Orlandi SP (2010). Validation of a Portuguese version of patient-generated subjective global assessment. Brazilian J Clin Nutr.

[35] World Health Organization. Health Topics: Ageing and Life Course. World Health Organization; 2017. Accessed July 20, 2020. Available: http://www.who.int/topics/ageing/en/.

[36] Botsen D, Ordan MA, Barbe C, Mazza C, Perrier M, Moreau J (2018). Dynapenia could predict chemotherapy-induced dose-limiting neurotoxicity in digestive cancer patients. BMC Cancer.

[37] Husi H, MacDonald A, Skipworth RJE (2018). Urinary diagnostic proteomic markers for dynapenia in cancer patients. Biomed Rep.

[38] Hu WH, Cajas-Monson LC, Eisentein S, Parry L, Cosman B, Ramamoorthy S (2015). Preoperative malnutrition assessment as predictors of postoperative mortality and morbidity in colorectal cancer: an analysis of ACS-NSQIP. Nutr J.

[39] Steemburgo T, Averbuch NC, Belin CHS, Behling EB (2018). Hand grip strength and nutritional status in hospitalized oncological patients. Rev Nutr.

[40] Guerra RS, Fonseca I, Pichel F, Restivo MT, Amaral TF (2015). Handgrip strength and associated factors in hospitalized patients. JPEN J Parenter Enteral Nutr.

[41] Pereira AAC, Zaia RD, Souza GHG (2020). The correlation between hand grip strength and nutritional variables in ambulatory Cancer patients. Nutr Cancer..

[42] White JV, Guenter P, Jensen G, Malone A, Schofield M (2012). Malnutrition task force and the a.S.P.E.N. board of directors. Consensus statement: academy of nutrition and dietetics and American Society for Parenteral and Enteral Nutrition: characteristics recommended for the identification and documentation of adult malnutrition (Undernutrition). J Parenter Enter Nutr.

[43] Ryan AM, Prado CM, Sullivan ES, Power DG, Daly LE (2019). Effects of weight loss and sarcopenia on response to chemotherapy, quality of life, and survival. Nutrition.

[44] Lew CCH, Ong F, Miller M (2016). Validity of the adductor pollicis muscle as a component of nutritional screening in the hospital setting: a systematic review. ClinNutr ESPEN.

[45] Gonzalez MC, Pureza Duarte RR, Orlandi SP, Bielemann RM, Barbosa-Silva TG (2015). Adductor pollicis muscle: a study about its use as a nutritional parameter in surgical patients. Clin Nutr.

[46] Valente KP, Silva NMF, Faioli AB (2016). Thickness of the adductor pollicis muscle in nutritional assessment of surgical patients. Einsten..

[47] Poziomyck AK, Corleta OC, Cavazzola LT (2018). Adductor pollicis muscle thickness and prediction of postoperative mortality in patients with stomach cancer. ArqBrasCirDig..

[48] Cederholm T, Barazzoni R, Austin P (2017). ESPEN guidelines on definition and terminology of clinical nutrition. Clin Nutr.

[49] Ordan MA, Mazza C, Barbe C (2018). Feasibility of systematic handgrip strength testing in digestive Cancer patients treated with chemotherapy: the FIGHTDIGO study. Cancer..

[50] Moreau J, Ordan MA, Barbe C (2019). Correlation between muscle mass and handgrip strength in digestive cancer patients undergoing chemotherapy. Cancer Med.

[51] Arvandi M, Strasser B, Meisinger C (2016). Gender differences in the association between grip strength and mortality in older adults: results from the KORA-age study. BMC Geriatr.

